# Study design flaws and statistical challenges in evaluating fertility treatments

**DOI:** 10.1530/RAF-21-0015

**Published:** 2021-06-17

**Authors:** Jack Wilkinson, Katie Stocking

**Affiliations:** 1 Centre for Biostatistics, Division of Population Health, Health Services Research and Primary Care, Faculty of Biology, Medicine, and Health, University of Manchester, Manchester, UK

**Keywords:** assisted reproduction, reproductive medicine

## Abstract

Health interventions should be tested before being introduced into clinical practice, to find out whether they work and whether they are harmful. However, research studies will only provide reliable answers to these questions if they are appropriately designed and analysed. But these are not trivial tasks. We review some methodological challenges that arise when evaluating fertility interventions and explain the implications for a non-statistical audience. These include flexibility in outcomes and analyses; use of surrogate outcomes instead of live birth; use of inappropriate denominators; evaluating cumulative outcomes and time to live birth; allowing each patient or couple to contribute to a research study more than once. We highlight recurring errors and present solutions. We conclude by highlighting the importance of collaboration between clinical and methodological experts, as well as people with experience of subfertility, for realising high-quality research.

**Lay summary:**

We do research to find out whether fertility treatments are beneficial and to make sure they don’t cause harm. However, research will only provide reliable answers if it is done properly. It is not unusual for researchers to make mistakes when they are designing research studies and analysing the data that we get from them. In this review, we describe some of the mistakes people make when they do research about fertility treatments and explain how to avoid them. These include challenges which arise due to the large number of things that can be measured and reported when looking to see if fertility treatments work; failure to check whether the treatment increases the number of live births; failing to include all study participants in calculations;challenges in studies where participants may have more than one treatment attempt. We conclude by highlighting the importance of collaboration between clinical and methodological experts, as well as people with experience of fertility problems.

## Introduction

It is hopefully not too controversial to suggest that fertility interventions should be robustly tested before they are introduced into routine practice. In the absence of robust testing, it cannot be known whether treatment improves fertility outcomes, or even worsens them (Braakhekke *et al.* 2017*a*, Wang *et al.* 2020). The challenge is to distinguish the subtle effects of an intervention from the plethora of other factors, most of which are unknown, that determine whether or not a patient will successfully conceive and have a child. In principle, randomisation of a sufficient number of participants allows us to achieve this goal (Yusuf *et al.* 1984, Wilkinson *et al.* 2019*a*). It might be possible to discern larger effects (e.g. those which lead to a substantial improvement in live births) without randomisation. However, without randomisation, we usually can’t tell whether any differences in outcome are actually due to other things which we have not, perhaps cannot, measure and adjust for. Regardless of whether or not randomisation is used, the reliability of a study’s results is dependent on good research design and execution, as well as appropriate data analysis and interpretation. The purpose of this review is to draw attention to some common pitfalls when using quantitative methods to evaluate fertility interventions.

Before we begin, we note that the following discussion is far from comprehensive. We have restricted our focus to issues that arise due to the nature of subfertility and its therapies. There are many other methodological fallacies which commonly occur in biomedical research irrespective of the clinical context. We do not cover these here, but point readers to more general resources in the section titled Conclusions. We have also opted to focus on the evaluation of interventions, and so do not cover methodological issues relating to the development of diagnostic or prognostic tools. We caution the reader that different considerations apply in that context, and again we point to some resources at the end of the article. Finally, we acknowledge that we are not the first to write a review of statistical issues arising in fertility research, with earlier examples focussing on trials (Vail & Gardener 2003) or observational studies (Messerlian & Gaskins 2017, Dodge *et al.* 2020). We have neither selected nor avoided topics based on these previous review articles. Instead, we have selected topics which we believe are consequential and have tried to produce a clear exposition of each with a non-statistical audience in mind.

### Multiple treatment stages introduce multiple methodological challenges

Many methodological challenges in fertility research arise due to the fact that the treatments under study are sequential in nature, involving multiple stages. For example, assisted reproduction typically involves ovarian hyperstimulation, fertilisation of the oocytes, culture of the resulting embryos, and selection of the best for transfer. Surplus embryos might be frozen and transferred in subsequent cycles. Even natural conception, involving no additional medical care, is typically evaluated over a period of several months, with multiple opportunities to get pregnant. Multistage treatments of this sort present a variety of options to the researcher when deciding both what to measure and who to measure it in. As we will explain, however, many of these options do not represent methodologically sound strategies.

### The problem of many outcomes

The performance of multistage-assisted reproductive treatments can be quantified at each stage. For example, the response to ovarian hyperstimulation, the number and quality of embryos obtained following fertilisation and culture, success of the embryo transfer, the outcome of pregnancy and health of any resulting offspring can all be measured and reported in a study involving *in vitro* fertilisation (IVF) (Heijnen *et al.* 2004). Accordingly, a recent review of outcome reporting in infertility RCTs found that many different outcomes (361 numerators and 87 denominators, resulting in 815 distinct combinations) appear in the literature, with a median (interquartile range) of 11 (7 to 16) reported per study (Wilkinson *et al.* 2016).

Reporting multiple outcomes of treatment in a study are not a problem in and of itself and can serve to give a fuller description of how an intervention influences live birth rates. Additionally, reporting metrics relating to safety, such as ovarian response to hyperstimulation and neonatal outcomes, is essential in order to permit the detection of potential harms. Nonetheless, the availability of an expansive menu of outcomes poses a threat to a study’s statistical validity if not handled appropriately. In particular, challenges arise when many statistical tests are performed (*multiple testing*) (Moher *et al.* 2010), or when results are selectively reported on the basis of the statistical test result (*selective outcome reporting*). (Furukawa *et al.* 2007, Al-Marzouki *et al.* 2008, Dwan *et al.* 2014) Unfortunately, the wide array of outcomes arising from fertility treatments means that there are lots of things to test and plenty of reporting options to choose from (Braakhekke *et al.* 2017*b*). Moreover, results may be reported for various subgroups of participants, compounding these problems. When multiple tests are performed, the chance of obtaining a statistically significant result under the null (e.g. a *P*-value < 0.05 when no effect exists) will be greater than 5% ( Farland *et al.* 2016, Roberts & Vail 2017). As a result, researchers may claim to have found evidence of an effect when in reality the play of chance represents a suitable explanation. Selective outcome reporting gives a misleading impression of the effect of a treatment in an individual study and also distorts the results of meta-analyses.

In order to protect against these concerns, it is advisable to have a single outcome measure prespecified as the primary outcome of the study (Moher *et al.* 2010). A statistical test of this outcome can then be used as the basis for the study conclusion. Statistical tests of secondary outcomes should also be prespecified and ideally limited in number, but even with these measures in place, they should be interpreted with caution. This principle introduces a tension between the desire to give a comprehensive account of the treatment process on the one hand and the desire to safeguard the credibility of statistical comparisons on the other (Althouse 2016). A pragmatic approach might be to fully report the procedural outcomes of treatment while restricting statistical testing to a small number of hypotheses relating to the postulated mechanism of the intervention effect; another approach is to adopt a hierarchical testing strategy (European Medicines Agency 2016). An example of a reasonably compelling finding arising from the analysis of a secondary outcome is the reduction in miscarriage using hyaluronan-based sperm selection for intracytoplasmic sperm injection (ICSI) (Miller *et al.* 2019). This example was prespecified, the number of tests performed was reasonably modest, and the corresponding test clearly indicated that the data were incompatible with the null hypothesis of no effect on miscarriage, with a *P*-value of 0.003.

Although strict prespecification of outcomes and analyses is a powerful strategy for maintaining the credibility of statistical inferences, outside of RCTs, this is not common practice in fertility research. The reasons for this might include a lack of awareness of the frailty of results obtained via data-driven statistical analyses (Simmons *et al.* 2011) and a lack of awareness of mechanisms and platforms for formal prespecification. Another reason might relate to the fact that a study is more likely to be published if it has a statistically significant finding (resulting in so-called publication bias(Easterbrook 1987, Easterbrook *et al.* 1991)). Since it is much easier to achieve statistical significance (albeit, of a spurious nature) via data-dredging than by performing a valid test of a prespecified hypothesis, the latter option is likely to appear less attractive. An innovative publication type, known as a Registered Report, represents a solution to this problem ( Chambers 2019, Chambers *et al.* 2015). In a Registered Report, authors submit a protocol for their research project to a journal for peer review, including the specific hypotheses to be tested, before the study has been initiated. The journal will make a decision regarding whether or not to publish the findings of the research at this stage. This means that publication is determined by the importance of the research question and the robustness of the methods rather than on the basis of the study results. The guarantee of publication removes the incentive for researchers to find (really, create the illusion of) statistically significant results, and the prospective declaration of hypotheses permits statistically sound testing to be conducted. This format has been adopted by some fertility journals (Wilkinson *et al.* 2019*b*) but is yet to gain a foothold in the field, despite the potential benefits for the evidence base.

### Diversity of definitions

The problems associated with variation in reporting in fertility research are exacerbated by the fact that multiple definitions are in use for some outcomes (Wilkinson *et al.* 2016). To illustrate, a review identified numerous definitions of biochemical pregnancy (23), clinical pregnancy (61), ongoing pregnancy (20) and live birth (7) (Wilkinson *et al.* 2016). This expands the array of reporting options. Concerningly, selective reporting of different versions of one outcome might be more difficult to detect than selection or omission of an outcome altogether. For example, suppose a research team finds an effect of a treatment on live birth if they include only birth events of at least 28 weeks gestation, but not if they include all events after 24 weeks gestation, and therefore, decides to use the former definition. This would be much less obvious than a study omitting live birth altogether.

An additional consequence of heterogeneity in outcome reporting is that it is difficult to compare and combine results of studies which report different outcomes or use different definitions of the same outcomes. The inability to include good-quality data in meta-analysis is a serious hindrance to progress in fertility research. This was highlighted by a recent review which found that meta-analyses of RCTs of fertility interventions were frequently uninformative, in part due to a lack of available data (Stocking *et al.* 2019). The recent publication of a core outcome set for infertility is intended to tackle outcome heterogeneity by specifying a minimum set of outcomes that should be routinely reported, using standardised definitions (Duffy *et al.* 2020). The success of this initiative is likely to depend on the commitment of journals to monitoring and enforcing adherence.

### Measuring success and surrogate outcomes

We have described the plurality of measures available for the purpose of evaluating interventions. How can the fertility researcher choose amongst them? The matter of measuring the performance of fertility treatments has been frequently debated over the past two decades (Heijnen *et al.* 2004, Min *et al.* 2004, Abdalla *et al.* 2010, Wilkinson *et al.* 2017, Goodman *et al.* 2020). Since people undertake fertility treatments with the goal of having a child, it is generally agreed that live birth is the most appropriate measure for the purpose of evaluating whether an intervention is clinically effective (Harbin Consensus Conference Workshop Group 2014). As such, live birth (Duffy *et al.* 2020) should generally be used as the primary outcome in studies aiming to evaluate clinical effectiveness (although Braakhekke and colleagues (Braakhekke *et al.* 2014) have made a reasonable argument for viable pregnancy of 12 weeks duration, on the grounds that pregnancy losses after this time are low).

However, we would emphasise that primary outcomes other than live birth are likely to be more appropriate when the objective is not to evaluate clinical effectiveness. For example, where an experimental intervention has a clear, postulated mechanism of effect, it can be more efficient and more ethical to design a study to test that mechanism before moving to a definitive clinical evaluation. To illustrate, consider a hypothetical novel ovarian stimulation protocol intended to increase the number of oocytes retrieved in predicted poor responders in IVF. Far fewer participants would be required to conduct a well-powered study if number of oocytes, rather than live birth, were used as the primary outcome. If the study ruled out an effect on oocyte yield, there would be no need to proceed to a larger study evaluating live birth, preventing the needless recruitment and allocation of many women to receive an ineffective treatment. A related idea that has been proposed in other clinical fields and which warrants investigation in fertility is to use upstream (or surrogate) outcomes as early indicators of futility during a trial. This involves using the data accrued up to that point to predict whether the trial is likely to identify a benefit of treatment. If not, we can stop the trial. Using a surrogate outcome, rather than waiting for live birth data to become available, would speed up this process (Bratton *et al.* 2013, Friede *et al.* 2020).

Nonetheless, it is important to note that the converse does not hold; while failure to improve a well-selected surrogate outcome might be sufficient for the purposes of deserting an intervention, improvement in a surrogate cannot necessarily be taken as proof that the overall clinical outcome (in fertility, usually live birth) would also be improved. Illustrating the point, Svensson and colleagues compiled a list of drugs which had been approved on the basis of a surrogate endpoint but were later shown to be deleterious (Svensson *et al.* 2013). Nonetheless, endorsing a treatment on the basis of a surrogate outcome appears to be a common fallacy in fertility research. For example, reductions in total fertilisation failure (TFF) are used as justification for using ICSI for non-male factor infertility. However, it is not clear that any advantage in terms of fertilisation translates to improvements in a live birth (Van Rumste *et al.* 2003). Caution would be required even if the evidence for ICSI reducing TFF in non-male factor infertility was robust. In reality, some of the trials indicating an improvement in TFF are within-person designs, where each participant’s oocytes have been randomly divided into ICSI and IVF groups. These designs will exaggerate improvements in TFF compared to clinical practice because the oocyte pools under consideration are half the size.

Surrogate outcomes can be seductive, particularly when there is evidence of correlation between the surrogate and the overall outcome. For example, studies investigating the ‘optimal’ number of oocytes following ovarian stimulation on the basis of correlation with live birth (Law *et al.* 2019, 2021, Sunkara *et al.* 2011) are sometimes misinterpreted as showing that achieving this optimum will maximise the chance of treatment success. But this doesn’t follow because a correlation between oocytes and live birth does not actually tell us how changing the number of oocytes changes the chance of having a baby. Accordingly, the observed correlation cannot be interpreted as evidence in favour of a treatment strategy which involves aiming for this optimal oocyte yield. In fact, the data would be consistent with (which is not to say *supportive of*) a disadvantage of this strategy; perhaps, the strategy would adversely impact the receptivity of the uterine environment in fresh transfers, reducing live births (for example). The effectiveness (and safety) of the strategy would require evaluation in a suitable comparative study.

The relationships between surrogate outcomes and live birth in fertility research appear to require further study. Some surrogate outcomes may indeed be *valid*, meaning that treatment effects on the surrogate reliably predict treatment effects on live birth. Established methodology exists to investigate the validity of surrogates (Bujkiewicz *et al.* 2016, Ciani *et al.* 2017).At present, effects on surrogate outcomes are used to advertise add-on treatments on IVF clinic websites (Van de Wiel *et al.* 2020, Lensen *et al.* 2021). Both patients and providers might be unclear about what this does or doesn’t mean for the add-on’s effect on live birth, and in general, we would caution against counting chickens before they hatch.

### Choosing denominators

A further complication introduced by multistage treatments relates to the possibility of using different denominators when calculating outcome measures. For example, in ART, live birth can be calculated with a variety of denominators, including per cycle started, per oocyte collection, and per embryo transfer. Debates around the choice of denominator often revolve around relevance to patients and clinicians; for example, it might be argued that live birth per cycle started is usually more relevant than live birth per transfer procedure, because the former incorporates any effect the intervention has on the likelihood that a transfer will take place. To illustrate, preimplantation genetic testing for aneuploidy (PGT-A) might appear superior to morphological embryo selection when success rates are calculated per transfer procedure but not when they are calculated per cycle started (or for that matter, per oocyte retrieval), because PGT-A reduces the chance that a transfer procedure will take place (Homer 2019, Munne *et al.* 2019). Proponents of PGT-A might respond that the per embryo transfer denominator is nonetheless relevant because it answers the question of whether treatment improves outcomes in women who undergo a transfer procedure.

**Figure 1:**
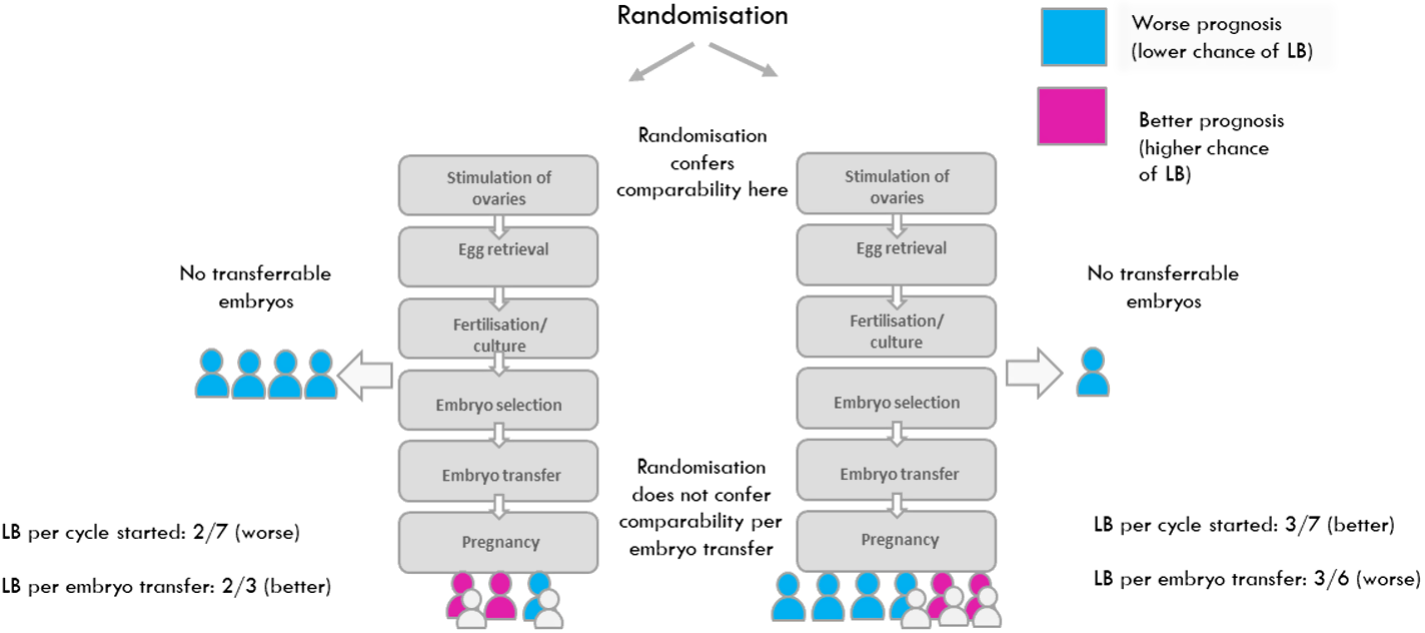
An illustration of why analysis ‘per embryo transfer’ does not provide a valid estimate of a treatment’s effect.

While the relevance of the research question to stakeholders is a critical consideration, an equally important concern is whether the methods employed actually answer that question. And in general, using an event which occurs after the experimental intervention has been administered to create the denominator (in this example, embryo transfer occurs after PGT-A) will not provide a valid assessment of the intervention effect. The problem is easiest to understand in the context of RCTs but applies to observational studies also. In an RCT comparing two interventions, randomisation confers comparability of the groups; while differences in patient characteristics are inevitable, the fact that they have arisen by chance allows for valid inferences to be drawn. When we use an event which occurs after randomisation as the denominator, we restrict the analysis to a post-randomisation (or ‘improper’) subgroup of participants (Yusuf *et al.* 1991, Hirji & Fagerland 2009). An example would be the subgroup of participants who undergo an embryo transfer procedure in a trial where randomisation took place earlier in the treatment process. However, while randomisation confers comparability of treatment arms in the full randomised cohort, this is not true for the post-randomisation subgroup. On the contrary, whenever treatment influences inclusion in the subgroup (for example, by affecting the likelihood of undergoing an embryo transfer) we expect patient characteristics to systematically differ between treatment arms, essentially a selection bias. Differences in outcome between arms in the subgroup will then reflect any differences in prognosis due to these characteristics and do not represent the effect of treatment. Accordingly, as illustrated in Fig. 1, an intervention could appear more effective ‘per embryo transfer’ simply by making it more difficult to proceed to a transfer procedure, thereby eliminating patients of worse prognosis from consideration. This would be an unusual interpretation of the notion of therapeutic benefit, presenting a challenge to the idea that this analysis is more relevant to patients.

Proponents of PGT-A might respond that Fig. 1 is not a fair representation of what happens when this intervention is used. In Fig. 1, the implication is that some of the people who didn’t undergo embryo transfer would have had a baby had they done so. By contrast, proponents may say that the advantage of PGT-A is not that it improves live birth rates, but rather that it reliably predicts failed embryo transfers, such that it prevents futile transfers and spares patients the experience of miscarriage. This framing implies that the prediction of transfer failure is near perfect, that PGT-A could not influence an embryo’s chances of implanting or being carried to term, and that there exist patients who would be willing to pay to reduce the chance of miscarriage without improving (and potentially decreasing) their chance of live birth. It is also curious that if the primary motivation for PGT-A is not to improve live birth but to reduce miscarriage, that RCTs of PGT-A have been designed to demonstrate superiority with respect to live birth rates. A more appropriate design to demonstrate the claim would evaluate noninferiority of PGT-A with respect to live birth while demonstrating a reduction in miscarriage per woman randomised. A noninferiority study aims to show that the intervention is not materially worse than the control (often the standard treatment).

These considerations do not imply that ‘per cycle started’ should always be used as the denominator. In many situations, an alternative denominator might be preferred, provided that a post-intervention denominator is avoided. As a general rule, there are advantages of beginning follow-up (in an RCT, performing randomisation) of participants as close to the delivery of the intervention under study as possible. For example, RCTs of intrauterine administration of hCG around the time of embryo transfer have randomised participants before the start of treatment, on the day of oocyte collection, and on the day of embryo transfer (Craciunas *et al.* 2018). Randomising on the day of embryo transfer reduces the scope for randomised participants to drop out from the study before receiving the experimental intervention, allowing for a clearer interpretation of any observed differences in outcome and reducing the required sample size. The randomised woman should then be used to form the denominator for live birth.

### Analysing infant outcomes *(sometimes there are no good denominators)*

An important question when evaluating fertility interventions is whether and how they affect the health of offspring. This introduces some difficult statistical questions because by definition infant outcomes are only defined for the subset of participants who have a baby, and as we noted in the previous section, restricting comparisons to a post-intervention subgroup is best avoided. For example, an RCT considered the impact of using different embryo culture media in ART on the birthweights of infants (Kleijkers *et al.* 2016). The appropriate course of action here is the subject of debate, with some methodologists arguing that simple comparisons of outcomes in the subgroup of people having babies are most appropriate (Snowden *et al.* 2020) and others insisting that bespoke causal inference methods are required (Chiu *et al.* 2020). The former camp has criticised the assumptions required to use the methods endorsed by the latter, although we note that the simple approach also entails assumptions; for example, it appears to require that the participants are representative of the target population, which is not usually a requirement for valid assessment of a treatment (Bradburn *et al.* 2020). We acknowledge the controversy but cannot resolve the matter here. Software has recently been made available to assist researchers in thinking about this issue when designing and interpreting fertility trials where outcomes are only defined in a subset of participants (Wilkinson *et al.* 2020), and interested readers are advised to peruse the reference list of that article.

### Evaluating treatment outcomes over multiple treatment attempts

Because a minority of people undergoing treatment have babies on their first try, a course of fertility treatment often involves multiple attempts to conceive. Many fertility interventions are expected to influence not only the outcome of the next attempt but also any subsequent attempts. For example, in a trial of endometrial scratching for unexplained infertility, the intervention was delivered in the first 12 days of the menstrual cycle, and the effect on natural conception rates over three cycles was evaluated (Lensen *et al.* 2016). Indeed, some interventions will affect the number of attempts that can be made. For example, any intervention which increases the number of embryos available for freezing following one ovarian stimulation cycle in ART will increase the potential number of embryo transfers in one full cycle. And in some cases, the intervention under study is itself a strategy comprising multiple attempts. For example, a trial of intrauterine insemination (IUI) compared to IVF is being conducted which will compare outcomes following up to four cycles of IUI with those following one full cycle of IVF (Prentice *et al.* 2020).

As these examples illustrate, in many cases it will be important to evaluate patient outcomes following a course of treatment, rather than restricting focus to the initial attempt(Heijnen *et al.* 2004). Broadly speaking, there are two possible approaches to doing this. The first is to consider the effect of an intervention on *cumulative live birth rates* (CLBR), a term which refers to the proportion of patients having a live birth following some maximum number of treatment attempts or after a given period of follow-up time (Malizia *et al.* 2009). The second is to consider the effect on *time to live birth*, which refers to how long it takes to have a live birth (Sunkara *et al.* 2020). The two concepts are quite closely related, and in fact, CLBR may be (and often is) estimated using methods for time-to-event analysis.

One analytic challenge which is common to both approaches is the matter of handling participants for whom the outcome status at the end of the follow-up period is unknown. In a routinely collected health dataset, this might happen because the patient ceased treatment before having a baby (either due to their own wishes or at the recommendation of a clinician). In the context of a prospective study, outcome data will be missing whenever a participant is lost to follow-up before having a baby. Assumptions must be made about these participants. Historically, when calculating CLBR, it has been suggested to perform calculations under conservative and optimistic assumptions and to assume that the truth lies somewhere in between (Malizia *et al.* 2009, Stewart *et al.* 2011). The conservative scenario amounts to assuming that the people with missing outcome data did not have a baby. The optimistic scenario (which has been erroneously referred to as the ‘optimal’ scenario in some papers (De Neubourg *et al.* 2021)) amounts to assuming that people with missing outcome data are just as likely to have a baby as those with observed outcome data. This approach, of providing conservative and optimistic estimates of CLBR, is still used (De Neubourg *et al.* 2021). A variation on this strategy is to make different assumptions based on what is known about the reason for the missing outcome data. For example, we might assume that people who have missing outcome data because they ceased treatment of their own accord have similar outcomes to people for whom outcomes are observed while assuming that people who were advised to stop treatment due to poor prognosis do not have a baby (Verhagen *et al.* 2008). More recently, an inverse probability weighting approach has been described which attempts to adjust the estimate of CLBR according to the measured prognostic characteristics of patients with missing outcome data (Modest *et al.* 2018). Whether using inverse probability weighting or an alternative method (such as regression), adjustment for prognostic variables can improve the credibility of missing data assumptions (see White *et al.* 2011, 2012) for practical advice and further discussion of this point).

An important note is that much of the methodological discussion concerning handling of missing outcomes in CLBR has considered the case where we wish to estimate the CLBR in a single cohort; the preceding paragraphs are no exception. In this situation, the designations ‘conservative’ and ‘optimistic’ are appropriate. However, extra care needs to be taken when we wish to compare outcomes between study arms (as we do in any reasonable study of the effect of an intervention). Suppose, in a two-arm study, that many more people drop out and have missing outcome data in the control arm than in the treatment arm. Assuming that anyone with missing data did not have a baby would give conservative estimates of the CLBRs in the two arms but would not provide a conservative estimate of the relative effectiveness of the experimental treatment compared to the control. It is advised to carry out a primary analysis based on one set of plausible assumptions about missing outcome data and to conduct a range of sensitivity analyses to assess the robustness of the result to alternative assumptions (White *et al.* 2011).

A subtle point to consider when choosing which assumptions to make about participants with missing outcome data is whether we should be making an assumption about what their unobserved outcome, as a matter of fact, *is* (e.g. given that they have ceased treatment but may continue to have unprotected intercourse) or rather, what the outcome would have been(e.g. were they, contrary to fact, to have continued treatment) (Diggle *et al.* 2007). This depends on the specific question we are trying to answer. If we are interested in the effect of a treatment in practice (accepting, e.g. that people may prematurely cease a course of treatment for a variety of reasons), then we should make assumptions about the actual unobserved outcome in these participants. If instead, we are interested in the effect of treatment if adhered to (undergoing the full course of treatment as intended) then we must make assumptions about what the outcome would have been had they completed the treatment (Mallinckrodt *et al.* 2017). In the latter case, it would be necessary to treat the outcomes of participants for whom outcomes were observed but who did not complete treatment as though their outcomes were missing for the purpose of analysis.

While cumulative outcomes are frequently relevant in the study of fertility interventions, recent reviews suggest that relatively few RCTs report these (Wilkinson *et al.* 2016, Kemper *et al.* 2020). Funding restrictions might be cited here, although there are counterexamples where follow-up over 12 months has been funded (Prentice *et al.* 2020). One final unresolved challenge relates to the current variation in defining and calculating CLBR (Maheshwari *et al.* 2015).

### Errors in the analysis of time to live birth

In recognition of the fact that fertility treatment is often emotionally and financially burdensome, there is a current interest in identifying treatments and treatment strategies that reduce the duration from treatment initiation to live birth (Sunkara *et al.* 2020). This focus on a patient-centred outcome is laudable. We would urge some caution, however. Although interest in time to live birth in subfertility is relatively new, methodology for the analysis of time-to-event data is not and is both well established and routinely used in other clinical fields, such as oncology. This methodology must be applied in the context of infertility with due regard to clinical expertise, in order to ensure that any analytic assumptions are reasonable, but we would stress that there is no need to reinvent the wheel. It is concerning to see analyses of time to live birth, and indeed commentaries asserting how it should be done, which appear to be unaware of the appropriate techniques and which consequently fall into some common, but fatal traps.

**Figure 2:**
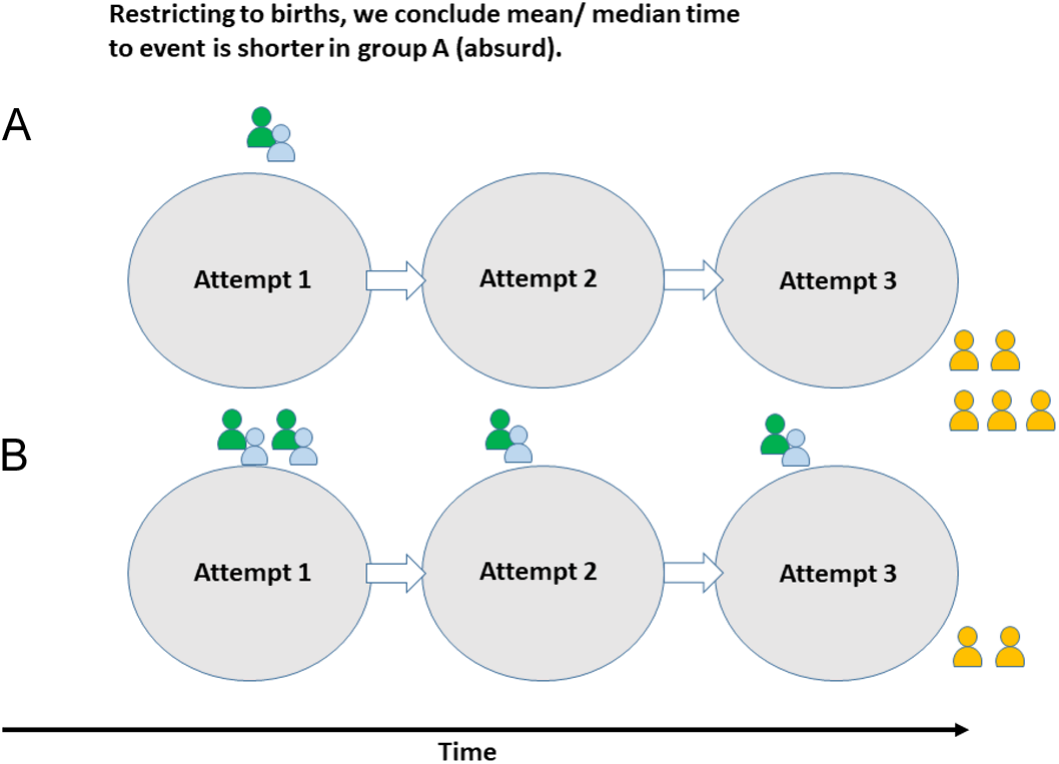
An illustration of why calculating ‘time to live birth’ using only people who had live births does not provide a valid estimate of a treatment’s effect.

One very natural but nonetheless completely erroneous approach, which has been used to suggest that PGT-A improves time to live birth, calculates the mean (or median) times using only the people who have had live births*.* The problems with this approach might not be immediately obvious, and indeed, this simple calculation might seem to be what is intended by the phrase ‘median time to event’. However, it isn’t. Figure 2 shows how this method can turn results on their head, suggesting an advantage of an inferior treatment. The figure shows participants in two treatment arms, A and B, undertaking up to three attempts at conception. The people coloured green have live births, and these are the only people included in the erroneous calculation. The people coloured yellow do not have live births after three attempts and are excluded from the erroneous calculation. Clearly, a patient can expect to have a live birth more quickly under treatment B than under treatment A. Under treatment A, most people (5/6) do not have a baby after three attempts, whereas under treatment B, most people (4/6) do. Yet the erroneous calculation will suggest that time to live birth is shorter under treatment A, even though more people have live births in the first attempt under B. One final absurd consequence of the erroneous calculation is that ‘time to live birth’ will depend on follow-up time; the longer the duration of the study, the longer the calculated median (mean) time to event will be.

Appropriate analyses instead include the follow-up time of all participants, regardless of whether they had a live birth or not. The methods to be applied in this context are those developed for *survival analysis*, so-called because the event of interest in other clinical fields is often mortality. The methods can be applied to any scenario where we are interested in the timing of an event, however. An exposition of survival analysis is well beyond the scope of the present article, and we restrict ourselves to some considerations regarding the application to fertility data. We have previously provided a very brief introduction in the context of fertility research in the supplementary material (Duffy *et al.* 2020). We recommend a short series of tutorial papers from the British Journal of Cancer (Bradburn *et al.* 2003*a*,b, Clark *et al.* 2003*a*,b) for a more thorough introduction, and the textbook by Collett (Collett 2015) for a comprehensive treatment of the subject.

What is the median time to event, if not the median time taken to have the event amongst those who have it? It is instead the time taken for 50% of the cohort to have the event of interest. In many fertility studies, we do not anticipate that as many as half of the participants will have a baby within the follow-up period. It might, therefore, be more appropriate to report the time taken for some smaller proportion of participants (e.g. 25%) to have live births. What about mean time to live birth? Although something like this could be calculated using appropriate statistical methods (Royston & Parmar 2011, 2013), these methods are yet to be widely adopted, and many research studies are conducted without sufficient statistical expertise to use them. Consequently, we believe that use of the phrase ‘mean time to live birth’ often serves as a red flag; it frequently indicates that the erroneous calculation, described above, has been performed. We also note that, although it is common to speak of ‘time to live birth’, as we have done in this article, this is probably not the most clinically appropriate metric. This is because the time to live birth could be shortened by increasing the number of preterm births, which is not a desirable characteristic of a treatment. Several solutions are possible. One option, which is included in a core outcome set for infertility, is to use ‘time to viable pregnancy leading to live birth’, where the event is defined as detection of pregnancy with one or more heartbeats on ultrasound, which subsequently results in a live birth (Duffy *et al.* 2020). Other possibilities are to define the event as ‘term live birth’ or to use the due date rather than the actual date of birth.

A final consideration is how to measure ‘time’ in these analyses. Possibilities are to use continuous time (e.g. weeks, days) or to treat time as discrete units, such as ‘number of attempts’ (Daya 2005, Missmer *et al.* 2011). The appropriate choice is likely to depend on the particulars of the research question, although comparing treatments in terms of ‘number of attempts’ might obfuscate any differences in actual time (Daya 2005). For example, if we compare a freeze-all strategy in ART to a conventional strategy of fresh, followed by frozen transfers, then we need to be mindful that two transfer attempts can be made in the conventional arm in the time it takes to conduct one in the freeze-all arm (Zaat *et al.* 2019).

### Multiple treatment periods per participant

In the preceding sections, we described the case where interventions influenced the outcome over multiple treatment attempts. In reality, we believe that this will usually be the case when assessing modern fertility treatments, particularly in the domain of ART. Our emphasis, therefore, differs slightly from previous authors who have described the case where an intervention is delivered repeatedly and may only affect the outcome of the next attempt. However, whenever this latter scenario does arise, the key statistical point is that the outcomes of repeated attempts undertaken by one individual (or couple) will tend to be more similar than those of several individuals (couples) (Vail & Gardener 2003, Roberts & Stylianou 2012, Missmer *et al.* 2011, Messerlian & Gaskins 2017, Yland *et al.* 2019, Dodge *et al.* 2020). It is a mistake to analyse repeated treatment attempts undertaken by the same individual (couple) as though they represented attempts of different individuals (couples), as has been observed to happen in some RCTs (Vail & Gardener 2003, Dias *et al.* 2008). In order to obtain valid statistical inferences, it is necessary to employ analytic methods capable of accommodating this correlation (Missmer *et al.* 2011, Roberts & Stylianou 2012, Yland *et al.* 2019). A similar error occurs when participants contribute multiple oocytes or embryos to a dataset, and these are analysed ignoring any relatedness (Vail & Gardener 2003). Further complexity arises due to the fact that the number of observations (attempts, oocytes, embryos) corresponding to each participant in the dataset is informative, requiring special treatment (Missmer *et al.* 2011, Yland *et al.* 2019). Performing appropriate analyses in this context requires a certain level of statistical competence, and since statistical experts are not the target of the current review, we do not go into any further detail here. Our recommendation to non-experts is to recognise the need for suitable expertise when conducting complex analyses; we direct statistical readers to the references appearing here and also to a review of methods for handling informative cluster size (Seaman *et al.* 2014).

Finally, we note that there is some controversy about the role of clinical trial designs which allow participants to be treated (and have an outcome assessment) on multiple occasions in the context of fertility research. These include crossover trials, in which participants are randomly allocated to receive each of the investigational and control interventions in a particular sequence (Senn 2002) and re-randomisation designs, in which participants are permitted to enter the study and be randomised to a treatment arm more than once (Kahan *et al.* 2015, 2018). If these designs are valid, they could reduce required sample sizes and improve recruitment to fertility RCTs, which would be a considerable benefit given the modest sizes of trials in the field (Stocking *et al.* 2019). The controversy arises because participants having a live birth typically do not proceed to receive further study treatment for subfertility, meaning, for example, that anyone having a live birth in the first period of a crossover trial would not proceed to the second period. It has been suggested both that valid inference is (Makubate & Senn 2010, Budhram *et al.* 2019) and is not (Daya 1993, Vail & Gardener 2003) possible under these circumstances. One practical consideration when applying these designs to fertility, which we have not seen discussed elsewhere, is that the outcome of previous treatment attempts will frequently be used to tailor the treatment delivered in a subsequent attempt. In ART, for example, the response to ovarian stimulation in a previous cycle is often used to modify the gonadotrophin dose and stimulation protocol in the next, and ICSI rather than IVF might be used if there were previous fertilisation issues. This might plausibly introduce a so-called carryover effect, which could be fatal to the validity of the crossover design, and consideration should be given to whether this sort of tailoring could be ethically prohibited. We speculate that tailoring on the basis of previous outcomes could also present a challenge for re-randomisation designs, if it influences the treatment effect in such a way that it is no longer constant each time a participant is randomised (Kahan *et al.* 2015). Careful consideration of this point in relation to the study intervention would be necessary. At least one RCT employing re-randomisation in ART is underway at the time of writing (Bhide *et al.* 2020).

## Conclusions

We have described some common methodological and statistical challenges arising in comparative effectiveness research in fertility. However, the issues we describe here are really just the tip of the iceberg. Various resources are available describing good statistical practice for biomedical research, but the quality is variable, with some making unfortunate errors. We recommend the *Statistics notes* series published by The BMJ (https://www.bmj.com/specialties/statistics-notes ) and textbooks by Altman (Altman 1999) and Bland (Bland 2015) for accessible discussions of many topics. As we noted in the introduction, different statistical and design considerations apply to prognostic and diagnostic research. An excellent collection of resources relating to prognostic research, including the development of clinical prediction models, is available at https://www.prognosisresearch.com/.

Ultimately, we emphasise that medical statistics, which we take to include research design, is a deep, diverse and difficult subject, which can be studied for many years without achieving anything close to mastery. While we hope that this review will help non-statisticians to appreciate some recurring but consequential statistical errors, the best way to improve individual studies is to seek statistical input at an early stage. Noting the possible conflict of interest, we, therefore, advise researchers to include methodologists as collaborators where possible. This will frequently require that their contribution can be paid for since the salaries of many statisticians in academic departments are supported by grant income. Careful, thoughtful data analysis takes time, and while inclusion on a research output is nice (and where a substantial contribution has been made, warranted) the currency of co-authorship isn’t acceptable as a salary for a key collaborator. Conversely, we stress that good medical research requires clinical as well as methodological expertise. We encourage applied statisticians to improve their own domain knowledge, as doing so facilitates discussion with clinical colleagues and helps to uncover methodological pitfalls which would otherwise be obfuscated by medical complexity. Indeed, statisticians who are not familiar with the field of fertility may not be alert to all of the methodological challenges we have described here. A meticulous dialogue between collaborators is, therefore, crucial for good study design; a last-second power calculation by email is not enough. Respectful collaboration between clinicians, methodologists, and partners with lived experience will remain the winning formula for useful fertility research.

## Declaration of interest

JW declares that publishing in peer reviewed journals is likely to benefit his career.

## Author contribution statement

Both authors wrote the manuscript and approved the final version.
